# Complete Genome Sequence of the Autotrophic Acetogen *Clostridium formicaceticum* DSM 92^T^ Using Nanopore and Illumina Sequencing Data

**DOI:** 10.1128/genomeA.00423-17

**Published:** 2017-05-25

**Authors:** Michael M. Karl, Anja Poehlein, Frank R. Bengelsdorf, Rolf Daniel, Peter Dürre

**Affiliations:** a Institut für Mikrobiologie und Biotechnologie, Universität Ulm, Ulm, Germany; b Genomic and Applied Microbiology and Göttingen Genomics Laboratory, Georg-August University of Göttingen, Göttingen, Germany

## Abstract

Here, we report the closed genome sequence of *Clostridium formicaceticum*, an Rnf- and cytochrome-containing autotrophic acetogen that is able to convert carbon monoxide to acetate using the Wood-Ljungdahl pathway. The genome consists of a circular chromosome (4.59 Mb).

## GENOME ANNOUNCEMENT

*Clostridium formicaceticum* DSM 92^T^ was first isolated in 1970 from sewers and ditches near Göttingen, Germany (1). It is described as a Gram-negative, obligate, anaerobic, mesophilic, motile, rod-shaped, and spore-forming organism with homoacetate fermentation during the exponential growth phase and additional formate production in the stationary growth phase (1). Unlike the close relative *C. aceticum*, *C. formicaceticum* is not able to grow at the expense of H_2_ + CO_2_, but chemoautolithotrophic growth was observed using CO + CO_2_ (2). Furthermore, the presence of cytochrome and menaquinone was demonstrated in this organism (3).

Extracted DNA was prepared for sequencing following the protocol for 1D genomic DNA sequencing for the MinION device using SQK-LSK108. Prior to the end-repair and dA-tailing step, the DNA was sheared using a g-TUBE (Covaris, Woburn, MA, USA) with an Eppendorf MiniSpin centrifuge (Eppendorf AG, Hamberg, Germany) at 5,500 rpm and repaired using NEBNext FFPE RepairMix (New England Biolabs, Ipswich, MA, USA). The library was loaded on a SpotON Flow Cell Mk I (R9.4) and sequenced using MinKOWN version 1.3.30. Raw data were base-called using Metrichor Desktop Agent version 2.43.1. The assembly was performed using the Canu version 1.4 assembler and resulted in six contigs (>10,000 bp) with an average coverage of 38-fold (4). In addition, isolated DNA was used to generate Illumina shotgun paired-end sequencing libraries, which were sequenced with a MiSeq instrument and the MiSeq reagent kit version 3, as recommended by the manufacturer (Illumina, San Diego, CA, USA). Quality filtering using Trimmomatic version 0.36 (5) resulted in 2,247,002 paired-end reads. SPAdes genome assembler software version 3.10.0 (6) was used for Illumina only and Illumina/Nanopore hybrid assemblies, resulting in 88 and 12 contigs (>500 bp), respectively. All assemblies were combined employing Gap4 version 4.11 software of the Staden package (7) and using the available, but unpublished, genome sequences of *C. formicaceticum* ATCC 27076 as the reference (CP017603). The closed genome of *C. formicaceticum* consists of a circular chromosome (4.59 Mb) with an overall G+C content of 35.53%. Automatic annotation and identification of rRNA and tRNA genes were performed using the Prokka software tool (8). The closed genome contained 33 rRNAs, 134 tRNAs, 3,062 protein-encoding genes with a predicted function, and 1,124 genes coding for hypothetical proteins. The analysis of the genome revealed that all genes were involved in the methyl and carbonyl branch of the Wood-Ljungdahl pathway. These genes form one cluster showing the same arrangement as found in *C. aceticum* and other clostridial autotrophic acetogens (9). Furthermore, all genes necessary for an Rnf complex were found. Genome analysis also revealed the genes for cytochrome synthesis, similar to *C. aceticum*. The inability of *C. formicaceticum* to use H_2_ + CO_2_ may be related to the absence of certain hydrogenases. Compared to *C. aceticum*, one hydrogenase gene cluster (CACET_c35700 to CACET_c35750) is missing in the sequenced genome.

### Accession number(s).

This whole-genome shotgun project has been deposited at DDBJ/EMBL/GenBank under the accession number CP020559.
